# Cost-effectiveness of active tuberculosis screening among high-risk populations in low tuberculosis incidence countries: a systematic review, 2008 to 2023

**DOI:** 10.2807/1560-7917.ES.2024.29.12.2300614

**Published:** 2024-03-21

**Authors:** Nino Gogichadze, Arnau Sagrera, José Ángel Vicente, Joan-Pau Millet, Francesc López-Seguí, Cristina Vilaplana

**Affiliations:** 1 Unitat de Tuberculosi Experimental, Germans Trias i Pujol Research Institute and Hospital (IGTP-HUGTIP), Badalona, Catalonia, Spain; 2 Universitat Autònoma de Barcelona (UAB), Cerdanyola del Vallès, Catalonia, Spain; 3 Research Group on Innovation, Health Economics and Digital Transformation (INEDIT), Institut de Recerca Germans Trias i Pujol, Badalona, Catalonia, Spain; 4 Fundació Lluita contra les Infeccions, Germans Trias i Pujol Research Institute and Hospital (IGTP-HUGTIP), Badalona, Catalonia, Spain; 5 Centre de Recerca en Economia de la Salut (CRES), Universitat Pompeu Fabra, Barcelona, Catalonia, Spain; 6 Servei d’Epidemiologia, Agència de Salut Pública Barcelona (ASPB), Barcelona, Catalonia, Spain; 7 Centro de Investigación Biomédica en Red de Epidemiología y Salud Pública (CIBERESP), Madrid, Spain; 8 Direcció Clínica Territorial de Malalties Infeccioses i Salut Internacional de Gerència Territorial Metropolitana Nord de l’Institut Català de la Salut, Badalona, Catalonia, Spain; 9 Centro de Investigación Biomédica en Red de Enfermedades Respiratorias (CIBERES), Madrid, Spain; 10 Microbiology Department, Northern Metropolitan Clinical Laboratory, Hospital Universitari Germans Trias i Pujol, Badalona, Catalonia, Spain; * These authors contributed equally to the work and share the first authorship.

**Keywords:** Tuberculosis, screening, high-risk groups, low-incidence countries, cost-effectiveness, effectiveness, vulnerable groups

## Abstract

**Background:**

In countries with a low TB incidence (≤ 10 cases/100,000 population), active pulmonary tuberculosis (PTB) mostly affects vulnerable populations with limited access to healthcare. Thus, passive case-finding systems may not be successful in detecting and treating cases and preventing further transmission. Active and cost-effective search strategies can overcome this problem.

**Aim:**

We aimed to review the evidence on the cost-effectiveness (C-E) of active PTB screening programmes among high-risk populations in low TB incidence countries.

**Methods:**

We performed a systematic literature search covering 2008–2023 on PubMed, Embase, Center for Reviews and Dissemination, including Database of Abstracts of Reviews of Effects (DARE), National Health Services Economic Evaluation Database (NHS EED), Global Index Medicus and Cochrane Central Register of Controlled Trials (CENTRAL).

**Results:**

We retrieved 6,318 articles and included nine in this review. All included studies had an active case-finding approach and used chest X-ray, tuberculin skin test, interferon-gamma release assay and a symptoms questionnaire for screening. The results indicate that screening immigrants from countries with a TB incidence > 40 cases per 100,000 population and other vulnerable populations as individuals from isolated communities, people experiencing homelessness, those accessing drug treatment services and contacts, is cost-effective in low-incidence countries.

**Conclusion:**

In low-incidence countries, targeting high-risk groups is C-E. However, due to the data heterogenicity, we were unable to compare C-E. Harmonisation of the methods for C-E analysis is needed and would facilitate comparisons. To outline comprehensive screening and its subsequent C-E analysis, researchers should consider multiple factors influencing screening methods and outcomes.

## Introduction

Tuberculosis (TB) is one of the leading severe bacterial infections worldwide, with an estimated 1.3 million deaths in 2022 [1]. Detection and treatment of TB have been a priority for the European Union/European Economic Area (EU/EEA) countries. The World Health Organization (WHO) launched the End TB Strategy in 2015 and set a goal of reducing the number of TB cases worldwide by 80% by 2030 [2]. To achieve these goals, and according to the WHO guidelines, it is essential to implement strategies for the early detection of TB and prompt TB treatment [3]. Screening tests are done to detect individuals with latent TB infection (LTBI), active disease or those who may have an elevated risk of developing TB. Establishing clear criteria for defining the population groups that warrant screening is crucial. Although there are strong recommendations for systematic screening of specific groups for TB (e.g. people living with human immunodeficiency virus (HIV) (PLHIV), household and other contacts of TB patients, people in prisons and individuals with past and present silica exposure), the certainty of the evidence ranges from very low to moderate. Notably, the incidence of TB is high within these groups. Recommendations for systematic screening for TB in subpopulations with structural risk factors (e.g. urban people with lower incomes, people experiencing homelessness, communities in remote or isolated areas, indigenous populations, migrants, refugees, internally displaced persons and other vulnerable or marginalised groups with limited access to healthcare) remain conditional, with very low certainty of evidence [4]. In countries with a low incidence of TB (≤ 10 cases/100,000 population) [5], a significant proportion of the cases are detected in vulnerable and hard-to-reach populations such as migrants, some of them from high TB incidence (> 100 cases/100,000 population) [4] countries [6], thus challenging the detection and treatment of TB [5]. Information on other subgroups affected by TB is either not available or is insufficiently stratified for conclusions. Cultural and language barriers, as well as lack or difficulty in access to healthcare, might severely delay TB diagnosis [7]. These factors increase the probabilities of complications, sequelae and mortality and enhance community infections and transmission [8]. Therefore, community screening of active pulmonary TB (PTB) in these populations might be key in low-incidence countries.

The WHO guidelines propose that national health programmes adapt the recommendations to their context, and this involves the need to identify the most effective combinations of tools for conducting systematic TB screening [3]. When designing and implementing systematic screening in specific settings, one of the key factors to consider is the optimisation of both efficacy and cost-effectiveness (C-E). There is a need for further studies to improve the guidance on how these screening interventions should be used in low-incidence countries to reach maximum effectiveness and C-E [3]. However, only a few studies have been performed in these population groups [9]. Moreover, most studies are conducted in high-incidence countries and, therefore, are poorly scalable to low-incidence countries [10].

To assess which screening approaches were cost-effective, we aimed to review the evidence available on the C-E of active PTB screening among high-risk populations in countries with low TB incidence.

## Methods

### Overall approach

To structure the main question of this review, we used a patient/population, intervention, comparison and outcomes (PICO) framework [11,12]. Subsequently, we generated the question “What is the C-E of active PTB screening of high-incidence communities in low-incidence countries?” As a population, we chose high TB incidence groups in low-incidence countries, and as an intervention active PTB screening. Comparisons were between screening policies. The outcomes included were the results of the effectiveness of screening and the C-E of screening.

### Search strategy and selection criteria

We followed the Preferred Reporting Items for Systematic Reviews and Meta-Analysis (PRISMA) 2020 statement: an updated guideline for reporting systematic reviews [13].

To identify articles of our interest, we performed a literature search on PubMed, Embase, Center for Reviews and Dissemination, including Database of Abstracts of Reviews of Effects (DARE) and National Health Services Economic Evaluation Database (NHS EED), Global Index Medicus and Cochrane Central Register of Controlled Trials (CENTRAL) of articles published between 1 January 2008 and 31 December 2023. The following key terms were used: (“tuberculosis” OR “screening” OR “active screening”) AND (“cost-effective” OR “cost effective” OR “C-E” OR “cost-effectiveness” OR “cost effectiveness”) AND (“low-burden” OR “low burden” OR “low incidence” OR “low-incidence”) OR (“high-incidence communities”) OR (“Europe”).

We conducted searches on the specified websites (WHO, European Centre for Disease Prevention and Control (ECDC), Centers for Disease Control and Prevention (CDC), GreyNet Internation (https://www.greynet.org/), OAIster (https://www.oclc.org/en/oaister.html), ClinicalTrials.gov (https://clinicaltrials.gov/) to retrieve grey literature. Our search yielded one article that was a duplicate, identified through the literature search mentioned above. No language filter was applied.

Two investigators (AS, NG) independently conducted a title and abstract screening and full-text screening. Studies that met the criteria listed in Box were considered relevant.

Box **Inclusion and exclusion criteria in a systematic review of cost-effectiveness of active tuberculosis screenings among high-risk populations in low tuberculosis incidence countries, 1 January 2008–31 December 2023**

**Inclusion criteria:**

- Retrospective and prospective studies
- Articles where active PTB screening was performed in low-incidence countries in high-risk groups and effectiveness and C-E analyses were performed
- Articles where active PTB and LTBI screening were performed in in high-risk groups of low-incidence countries and effectiveness and C-E analyses were performed

**Exclusion criteria:**

- Articles concerning solely LTBI or extrapulmonary TB (EPTB) screening
- Systematic reviews, scoping reviews and meta-analyses
- Articles studying new screening or diagnostic tools
- Articles only studying contact tracing
- Lack of C-E analysis
- Incomplete data

### Data items and data extraction

Two researchers (NG and AS) reviewed the full texts of the selected articles and did not confirm or obtain additional data from the authors. All disagreements between investigators were resolved by discussion.

### Summary methods

Data were collected in accordance with the predetermined variables, which are provided in Supplement. The contents extracted were placed in tables without changing the original text. To address heterogeneity, we incorporated suboptions for the same variable identified in the final screened papers into the spreadsheet. We then conducted an analysis to determine which suboptions were present in each of the studies, as well as which were most identified and how they influenced the outcomes. All currencies in the text and the table were converted to 2023 Euros, as shown in Supplement (Spain was used as a reference country) using an online currency conversion tool https://eppi.ioe.ac.uk/costconversion/default.aspx.

We used Risk Of Bias In Non-randomised Studies - of Interventions (ROBINS-I) tool (https://sites.google.com/site/riskofbiastool/welcome/home?authuser=0) to evaluate the risk of bias in the studies included [14]. The findings are summarised in Table 1 in Supplement.

## Results

### Literature search findings

Our search yielded 6,318 articles from the searched databases and one from other sources (Figure). After duplicate removal, 5,638 articles were screened by title and abstract, 5,335 were excluded and 303 articles were sought for retrieval. We could not retrieve four articles. The full text of 299 studies was assessed, 290 were excluded for different reasons: out of scope (n = 169), LTBI-related (n = 59), high incidence settings (n = 27), no C-E (n = 14), review (n = 13), drug trial (n = 3), methods unclear (n = 3), published in 2000 (n = 1) or abstract (n = 1). The abstract provided was from an article included in the review. Consequently, the abstract was excluded. Nine articles were included in this review. We were unable to perform a meta-analysis, considering that the articles included in the review did not use common measures and used different methodological approaches.

**Figure:**
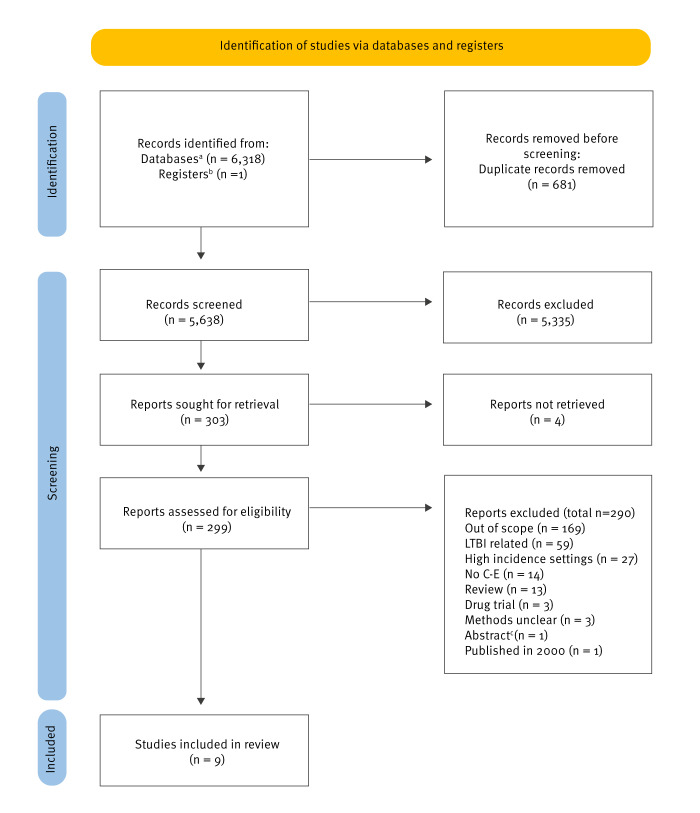
PRISMA flow diagram, systematic review of cost-effectiveness of active tuberculosis screening among high-risk populations in low tuberculosis incidence countries, 1 January 2008–31 December 2023

C-E: cost-effectiveness; LTBI: latent tuberculosis infection.

^a^ PubMed, Embase, Center for Reviews and Dissemination, including Database of Abstracts of Reviews of Effects (DARE) and National Health Services Economic Evaluation Database (NHS EED), Global Index Medicus and Cochrane Central Register of Controlled Trials (CENTRAL).

^b^ World Health Organization, European Centre for Disease Prevention and Control, Centers for Disease Control and Prevention, GreyNet Internation (https://www.greynet.org/), OAIster (https://www.oclc.org/en/oaister.html), ClinicalTrials.gov (https://clinicaltrials.gov/).

^c^ The identified abstract was of an article included in the review.

All the nine articles included were on active PTB cases in low-incidence countries in high-risk cohorts, such as people in prison, migrants, asylum seekers, youths in juvenile detention centres, nursing homes, people with substance use disorder, contacts of TB cases and isolated communities [15-23]. A summary of the articles reviewed is in Table 1. Table 2 provides a summary of the strengths and weaknesses of the included articles.

**Table 1:** Characteristics of studies included in a systematic review of cost-effectiveness of active tuberculosis screenings among high-risk populations in low tuberculosis incidence countries, 1 January 2008–31 December 2023 (n = 9)

Study	Country	Year	Screening method	Screened	Target population	Active PTB cases detected	Cost-effectiveness		
							ICER	Yes/No	Result
Pareek et al. [15]	UK	2013	Combination of CXR, no CXR, QFN-GIT, T-SPOT.TB	10,000	A hypothetical cohort of foreign-born immigrants	Cases of active TB over a period of 20 years^a^ (1) 73.4 (2) 71.7	Money per TB case saved	Yes	(1) Port of arrival CXR and QFN-GIT at 40/100,000 incidence; (2) Port of arrival CXR and T-SPOT.TB at 40/100,000 incidence. The associated ICERs for these strategies were EUR 68,941 and EUR 46,6360 per active TB case averted.
Cavany et al. [16]	UK	2019	TST/IGRA, CXR	NA	Contacts of PTB index cases	1-year perspective: No transmission: 45 cases averted,1 new infection: 62.4 cases averted, 2 new infections: 95.6 cases averted	Cost per QALY gained	Yes	Cost per QALY of screening the contacts of PTB cases was EUR 46,067/QALY in case of no transmission, EUR 31,941/QALY in case of r = 1 and EUR 19,713/QALY in case of r = 2.
Capocci et al. [17]	UK	2020	Questionnaire + TST/IGRA + CXR + Induced sputum	219	PLHIV	2	Cost per QALY gained	Yes	Strategies testing BA or people coming from countries with an incidence > 40/100,000 population with TST (with or without CXR) were inside the NICE C-E threshold. Single TST in BA EUR 15,772; Single TST in BA + MI: EUR 18,111; BA with single IGRA + CXR: EUR 28,403; Single IGRA in BA + MI: EUR 32,463.
Jit et al. [18]	UK	2011	CXR	NA	Individuals from drug treatment services, hostels or day centres for people experiencing homelessness or with lower incomes	1-year perspective: 16	Cost per QALY gained	Yes	The yearly cost of screening was EUR 614,235 ICER for screening was EUR 20,859 QALY.
Verma et al. [19]	CA	2013	TST, CXR	NA	People entering long-term care	4-year period: 4.5 cases	Cost per case (prevented)	No	The total cost of active PTB screening per 1,000 entrants was EUR 432,577. With ICER of EUR 443,562 for cost per case prevented.
Uppal et al. [20]	CA	2021	TST, past active PTB/LTBI history, CXR	1,650	Isolated community members	13	Cost per case estimated	Yes	One round of active screening in 2019 for one outbreak and in case of an outbreak every 3 years was cost saving. Biannual screening for outbreaks happening every 3 years and LTBI testing was cost-effective. All compared with no intervention and with the previous dominated strategy.
Smit et al. [21]	BE	2017	TST/IGRA + CXR	17,539	Asylum seekers, undocumented migrants in detention centres, other migrants from high-incidence countries, people in prison, contacts of active PTB patients and others	65	Cost per QALY gained	Yes	The average costs of the most cost-effective strategies were EUR 6,540 (follow-up of asylum seekers with CXR in case of abnormal findings), EUR 13,397 (screening of people in prison), EUR 16,097 (contact tracing) compared with no intervention.
Wahedi et al. [22]	DE	2020	Questionnaire, CXR, sputum culture	84,505 or 30,037 with < 50/100,000 incidence threshold	Migrants	Total: 73 From high-incidence countries: 58	Cost per case estimated	Yes	Screening of asylum seekers coming from countries with an incidence > 50/100,000 was cost-effective, with ICER of between EUR 13,409 and EUR 15,197 compared with no intervention.
Goscé [23]	IT	2021	Questionnaire, GenXpert MTB/RIF Ultra	3,787	Migrants	15	Cost per case estimated	Yes	The policy was cost-effective, with an ICER of EUR 2,993 compared with no intervention

BE: Belgium; CA: Canada; C-E: cost-effectiveness; CXR: chest X-ray; DE: Germany; EUR: euro; HIV: human immunodeficiency virus; ICER: incremental cost-effectiveness ratio; IGRA: interferon-gamma release assay; IT: Italy; LTBI: latent tuberculosis infection; MI: medium TB Incidence countries; MTB: *Mycobacterium tuberculosis*; NA: not applicable; NICE: National Institute of Health and Care; PLHIV: people living with HIV; PTB: pulmonary tuberculosis; QALY: quality-adjusted life year; r: Pearson correlation coefficient; QFN-GIT: QuantiFERON-TB Gold In-tube test; RIF: resistance to rifampicin; TB: tuberculosis; T-SPOT: an indirect test for the detection of effector T cells that respond to stimulation by *Mycobacterium tuberculosis* antigens; TST: tuberculin skin test; UK: the United Kingdom.

^a^ (1) Port of arrival CXR and QFN-GIT at 40 cases per 100,000 population; (2) port of arrival CXR and T-SPOT.TB at 40 cases per 100,000 population.

All currencies were converted to 2023 Euros (Spain was used as a reference country) using an online currency conversion tool https://eppi.ioe.ac.uk/costconversion/default.aspx.

**Table 2:** Strengths and weaknesses of studies included in a systematic review of cost-effectiveness of active tuberculosis screenings among high-risk populations in low tuberculosis incidence countries, 1 January 2008–31 December 2023 (n = 9)

Study	Strengths	Weaknesses
Pareek et al. [15]	Identification of optimal screening thresholds	Empirical data were available from a small sample size
	Final recommendations supported by real data	Assumptive rates of HIV, chemoprophylaxis acceptance and completion
	Suggested threshold incidence for screening	Model considered one generation of TB transmission
		Limited concurrent screening
Cavany et al. [16]	Proposed novel way to quantify effectiveness of contact tracing	Uncertain rate of transmission
	High-quality real data used	No analysis of the indirect impact of contact tracing on transmission within the population
		The infectious period of index patients was estimated based on self-reported symptomatic periods
Capocci et al. [17]	First reported study to model different testing strategies for LTBI, asymptomatic and symptomatic TB disease in PLHIV	Only 50% of approached patients agreed to participate
	Real data used	Difficulties in determining TB cases accurately in individuals with TST/IGRA undergoing ART was challenging
	Suggested threshold incidence for screening	
Jit et al. [18]	Not to overestimate benefit of the screening, analysis was performed using both favourable and unfavourable assumptions.	Lack or randomisation of managed and non-managed individuals, resulting in uncertainty of the outcomes
	Real data used	Secondary transmission was not considered in economic evaluation
		Likelihood of patients developing and transmitting drug resistance was not measured
Verma et al. [19]	Identified the risk of LTBI reactivation as the most influential variable in the analysis	Limited data on LTBI reactivation risk
		Lack of considering death before transmission, movement into and out of long-term care, environmental factors
Uppal et al. [20]	Highlighted the importance of involving communities in screening activities	Assumptive costs of LTBI and active TB case detection
	Emphasised on benefits of early case detection	Lack of age stratification, region-specific data for certain parameters
	Real data used	
Smit et al. [21]	Real data used	Contact investigation considered as an independent component
	Possible applicability of the results to other low-incidence countries	
Wahedi et al. [22]	Real data used	Uncertain rate of transmission
	Stratification of individuals	No data on MDR-TB prevalence
	Suggested threshold incidence for screening	The cost of overdiagnosis was assessed regarding CXR, with no examination of cost of confirmatory diagnosis or treatment initiation.
Goscé et al. [23]	Real data used	Did not use transmission dynamic model
		Using a “do-nothing” approach as a comparator was challenging because of incurred costs from late case detection
		Individuals were not followed up to assess effects of early detection or the consequences of failing to identify cases.

ART: antiretroviral therapy; CXR: chest X-ray; HIV: human immunodeficiency virus; IGRA: interferon-gamma release assay; LTBI: latent tuberculosis infection; MDR-TB: multidrug-resistant tuberculosis; PLHIV: people living with human immunodeficiency virus; TB: tuberculosis; TST: tuberculin skin test.

There were disparities in screening methods, the target population, defining C-E results and estimation of actual costs.

Eight of the nine studies used chest X-ray (CXR) as the primary screening tool for active PTB [15-22]. Two studies from Canada targeted isolated community members and long-term care entrants [19,20]. Four studies from the United Kingdom (UK) involved foreign-born immigrants, contacts of PTB index cases, people with substance use disorder, people experiencing homelessness, people with lower incomes and PLHIV as a target population [15-18]. Additionally, studies from Italy and Germany involved migrants, while the Belgian study focused on migrants as well as on asylum seekers, people in prison, contacts and other people who did not belong to any specific group [21-23]. Eight of the nine studies demonstrated that screening was cost-effective compared with no screening, the passive screening method or other alternatives [15-18,20-23].

### Cost-effectiveness results

Screening of PLHIV from Sub-Saharan Africa region or from countries with a medium TB incidence (MI) (> 40 cases/100,000 population) was found cost-effective and fulfilled the National Institute of Health and Care (NICE) threshold of EUR 22–33,000 [24]. Screening of people from Sub-Saharan Africa with interferon-gamma release assay (IGRA) and tuberculin skin test (TST) prevented the highest number of cases. Also, screening for active TB using CXR and TST could have been the most cost-effective method if the cost of CXR decreased [17].

Pareek et al. first performed active PTB and LTBI screening of 231 immigrants using IGRA or TST tests and CXR. Later, for the C-E analysis, the authors created a hypothetical cohort of 10,000 immigrants over a 20-year horizon with the aim of finding cost-effective strategies. Of the 53 included screening strategies of migrants from MI countries, port-of-arrival CXR combined with QuantiFERON-TB Gold In-Tube (QTF-GIT) and port-of-arrival CXR combined with T-SPOT.TB (https://www.tspot.com/) were cost-effective [15].

Cavany et al. performed C-E of screening contacts of TB cases in London. The static model used included index cases, their contacts, the average number of PTB cases in a year and hypothetical transmission rates (R = 0, R = 1, R = 2). When 2,790 PTB index cases were included and no transmission (R = 0), QALY was 39.9, costs incurred EUR 1,933,603.19 and incremental cost-effectiveness ratio (ICER) was 43.7. When the transmission rate was R = 1, the QALY was 56.3, costs EUR 1,802,624.06, ICER 1.63 and when R was 2, QALY was 43.7, costs EUR 31,941,233.29 and ICER was 18.7. Screening contacts of PTB cases was probably cost-effective at EUR 33,000 per QALY NICE threshold [16].

Another C-E study from London was performed using data from a mobile radiography unit – the Find and Treat service, which via targeting high-risk groups identified 16 active PTB cases per year. The number of people screened was not included in the publication. The screening costed EUR 614,235.68 with the ICER of EUR 21,000–30,000 per QALY gained, which is cost-effective according to the NICE threshold [18].

Uppal et al. compared no active screening, individual community-wide active screening strategy and community-wide screenings conducted every 2 years [20]. While both active screening methods were cost-effective, the individual community-wide active screening strategy was the most cost-saving.

The C-E of different screening strategies of people entering long-term care in Canada were simulated [19]. The cost of screening active PTB using CXR was EUR 432,577.67 per 1,000 people entering long-term care. To prevent one case, 1,266 individuals needed to undergo screening. The cost incurred per case averted was EUR 443,562.18, indicating that the screening was considered expensive.

Screening of migrants in first reception centres at Italian borders costed EUR 40,702.33 and a cost of a true positive case detected was EUR 2,712.97, lower than the median cost for detection of active PTB cases using the passive system in Italian hospitals (EUR 8,280.01) [23].

In Belgium, active screening of asylum seekers, contacts, undocumented immigrants, people in prisons and youth held in juvenile detention centres was performed. The total cost of all screenings performed was as expensive as the passive case findings [21]. However, examining the C-E of strategies on a case-by-case basis revealed screening of asylum seekers with an abnormal CXR finding at entry, systematic screening in prisons and contract tracing cost-effective. Screening individuals in juvenile detention centres or new immigrants and asylum seekers from high-incidence countries were not C-E.

Finally, three distinct publications have illustrated the importance of integrating the TB incidence rates from individuals' countries of origin into the C-E analysis: screening individuals from populations with TB incidences of > 50 cases per 100,000 population [22], as well as those with incidences < 50 cases per 100,000 population [22], along with populations where the incidence is > 40 cases per 100,000 [15,17].

## Discussion

The aim of this manuscript was to review active PTB screening programmes run between 2008 and 2023 in high-risk groups living in low TB incidence countries. Active PTB screening of high-risk groups in low-incidence countries may be C-E. These findings align with the systematic review by Greenaway et al. covering 2000–2016 [9]. In our review, CXR was an effective and cost-effective screening tool in eight studies [15-22], as in two studies included in the Greenaway et al. review [25,26]. The ICERs for cost-effective policies in our study were between EUR 15,000 and 30,000, which is a similar range as in the above-mentioned review [9].

Screening of PLHIV for TB is a well-established practice [1]. Though the level of evidence to screen for TB in people in prison is low, WHO strongly recommends the screening [3,27]. To systematically screen people with structural risk factors, WHO has issued conditional recommendations with low or very low certainty of evidence [3]. There is no more precise guidance on which migrants to target, the best timing to screen or the optimal threshold of TB incidence in the countries of origin. Greenaway at al. suggest screening individuals originating from countries with an incidence of 60 cases per 100,000 population, but two papers in our review indicate that this cut-off could even be lowered to 40 cases per 100,000 population [9]. We would like to highlight that return visits of migrants to their home countries might also be a noteworthy factor to consider. These visits could potentially lead to new infections, thus increasing the risk of developing active PTB. Hence, when implementing active TB screening activities, information on visits of migrants to their home country should be collected.

Six of the nine studies included in our review had co-authors from governmental bodies, pointing at the governments concerns on TB [15-18,20,21]. Public health-related research outputs should influence policymaking, and this influence should be assessed based on the level of policy impact. Available guidance does not address the specificities of particular settings, and evidence-based examples of how screening activities work in real setting are important to shape and adapt policies. Our study demonstrates that although still limited, the literature containing this information is growing. Publishing real-life screening activities can help others to design similar processes and to further adjust policies to the needs of the target settings.

Ideally, studies intending to conduct C-E analysis on screening programmes should be prospectively designed, as this allows for advanced planning of all aspects. However, this approach is expensive and not always feasible; thus, retrospective approaches are more common. While retrospective data may not be optimal, the information they offer can be highly valuable. Most of the studies in our review used retrospective data. Authors could draw conclusions applicable at least within their own countries and offer insights on implementing, conducting, discontinuing or sustaining existing screening activities. The evidence from this review highlights the advantage policymakers can gain by considering the C-E analyses of experiences of other countries even if they are retrospective studies. Although the data may be confined to a single screening strategy, comparing its costs to the no-screening policy can offer significant value for policymakers. With more data available, additional comparisons can be made, leading to more robust conclusions about the most cost-effective strategy.

Our review had some limitations. Besides few studies included, the data were heterogeneous. For a C-E analysis, it would be desirable to include as many variables as possible that directly affect the results, but this was not the case for the papers included. For instance, to estimate saved costs from prevented cases and possible adverse factors, the C-E analysis should include data on transmission rate and diagnostic delay. In summary, the cost calculations varied in the reviewed studies as some did not include treatment costs. The ICERs were calculated in various ways: cost per active PTB case detected, cost per case prevented or cost per QALY gained. The variables included were different. It was impossible to calculate possible savings as there were no data on diagnosis delay, transmission rate and patients lost to follow-up. Due to the scarcity of screening studies including C-E analysis, we were unable to provide strong recommendations, although some guidance has been drawn from the available evidence. Conducting a C-E analysis becomes challenging when multiple variables like the setting, approach, target population and screening method are included. Previous studies have not explored the interdependence of these variables and their influence on screening. Current limited available evidence on C-E underscores the need to develop guidance for the analysis methodology when implementing active PTB screening in the EU and other countries with low incidence. Such recommendations would greatly aid the development and enactment of national-level policies.

Regardless of the limitations described, our findings can be used to design comprehensive screening activities. It is important to consider the settings in which screening is performed, as screening policies need to be explicitly tailored to the characteristics and needs of the target population. For example, implementing TB screening at the borders-of-entry can be very beneficial to capture cases as early as possible, preventing severe outcomes for the individuals and the spread of the disease. Nonetheless, it is crucial to consider that newly arriving migrants in Europe often face poor living conditions and relocation, posing challenges to screening activities and the public health programmes need to overcome this. Implementing a screening process on the borders of the EU where most migrants enter, is pivotal [6]. Collaboration between countries will be mutually beneficial, generate a better understanding, more homogenised data and help implement better and ethical practices. Moreover, cultural factors cannot be overlooked, and screening activities should always consider the inclusion of key community leaders and organisations when designing and implementing the programme, as the individual willingness to participate in the population to be screened will significantly influence the success of the screening.

## Conclusion

Based on the findings spanning 16 years and the guidelines available, we have listed several recommendations for optimising study design for active screening programmes for TB in low-incidence countries. Firstly, to guide policymakers, C-E analysis should always be part of TB screening activities targeting high-risk groups in low TB incidence countries. Additionally, C-E studies should follow national economic evaluation guidelines in case they are available and consider the published WHO guidance. Moreover, the C-E analysis should encompass not only the expenses related to screening, but also those related to diagnosis and treatment. The transmission outcomes and how they affect the C-E of the interventions should also be calculated. If the transmission rate is high, early detection of a TB case will prevent secondary infections and illness and thus save costs. Finally, following the checklist outlined by the Consolidated Health Economic Evaluation Reporting Standards (CHEERS) will foster standardisation of reported data, facilitating comparability.

Implementing all these suggestions should allow further creation of recommendations for common use.

## Supplementary Data

155000 Supplementary Material
