# Supplementary material for: Cost-effectiveness of active tuberculosis screening among high-risk populations in low tuberculosis incidence countries: a systematic review, 2008 to 2023
Source: Euro Surveill. 2024 Mar 21;29(12):2300614. doi: 10.2807/1560-7917.ES.2024.29.12.2300614 (PMC11063676; doi:10.2807/1560-7917.ES.2024.29.12.2300614)
Supplement: Supplementary Material [file 23-00614_VILAPLANA_Supplement.pdf]

Supplementary material

This supplementary material is hosted by *Eurosurveillance* as supporting information alongside the article [Cost-effectiveness of active tuberculosis screenings among high-risk populations in low tuberculosis incidence countries: a systematic review], on behalf of the authors, who remain responsible for the accuracy and appropriateness of the content. The same standards for ethics, copyright, attributions and permissions as for the article apply. Supplements are not edited by *Eurosurveillance* and the journal is not responsible for the maintenance of any links or email addresses provided therein."

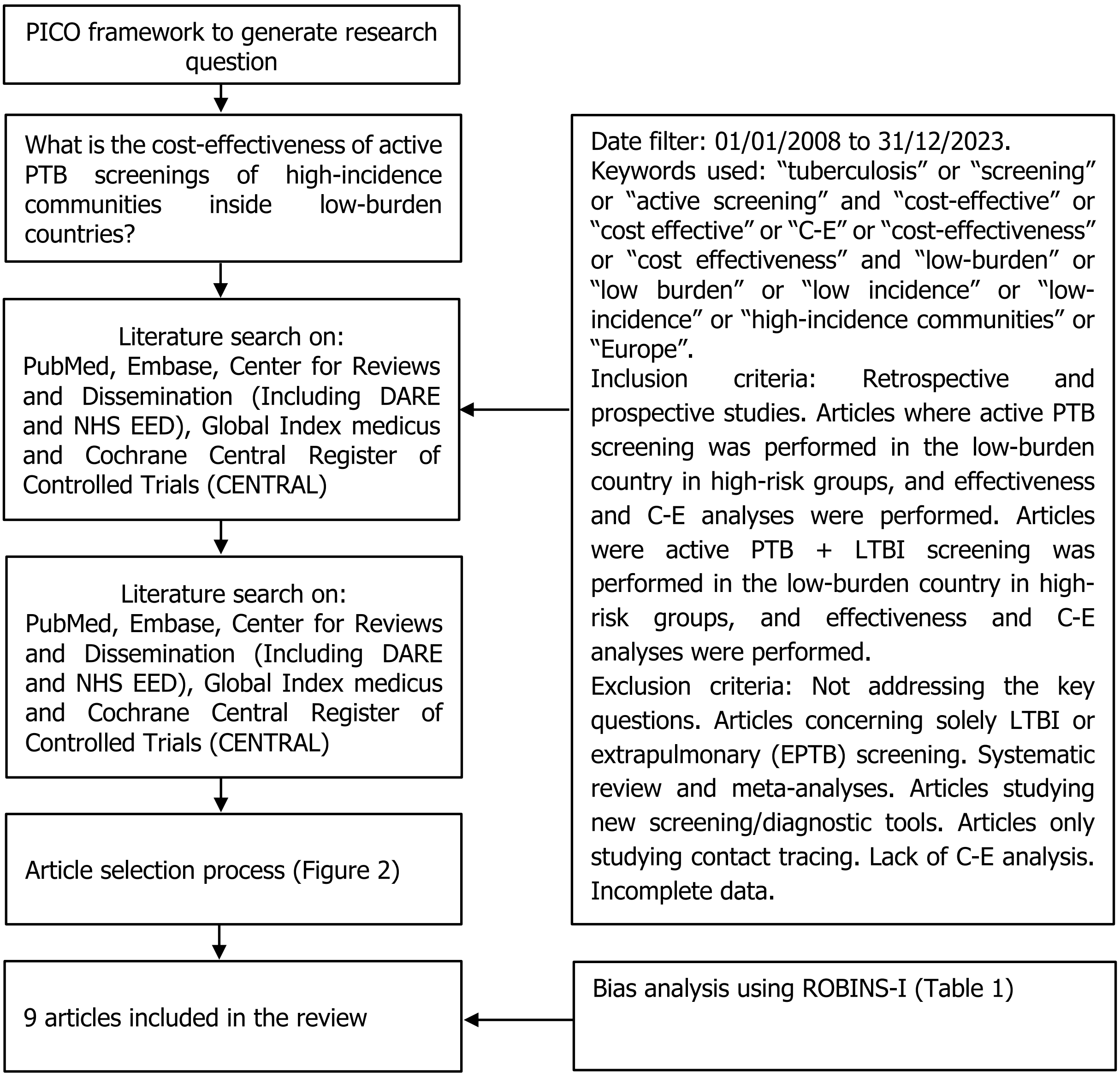

Figure 1: overall approach

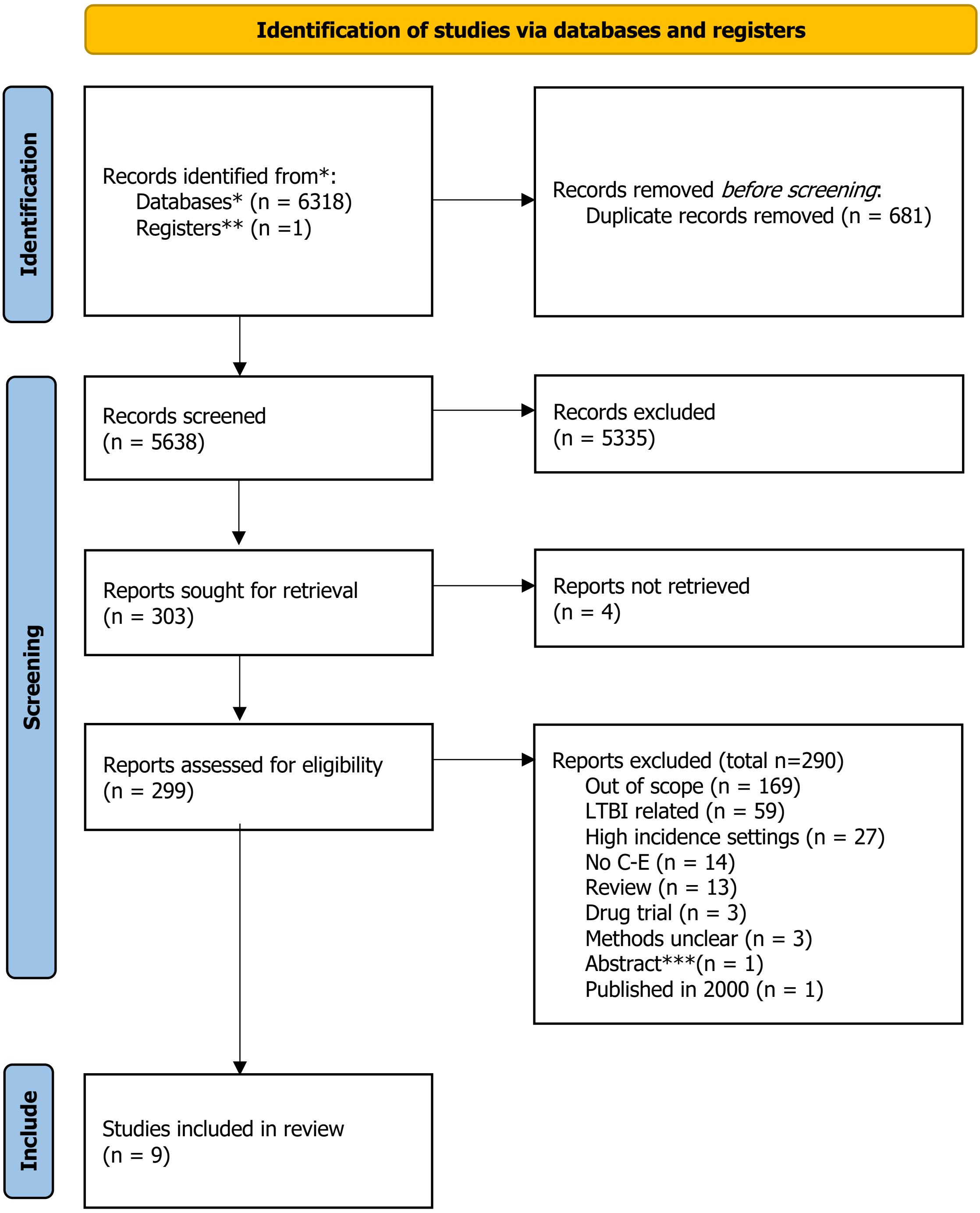

Figure 2
* PubMed, Embase, Center for Reviews and Dissemination (Including Database of Abstracts of Reviews of Effects (DARE) and NHS Economic Evaluation Database (NHSEED)), Global Index Medicus and Cochrane Central Register of Controlled Trials (CENTRAL).
** World Health Organization, European Centre for Disease Prevention and Control, Centers for Disease Control and Prevention, GreyNet Internation, OAIster, clinicaltrial.gov
***Identified abstract was for the artcile that was included in the review
LTBI: latent tuberculosis infection; C-E: cost-effectiveness

Data items and data extraction - VARIABLES

We reviewed the full texts of the articles of our interest and did not confirm or obtain data from the authors. All disagreements between investigators were resolved by discussion. To answer the question "What is the cost-effectiveness of active TB screenings for high-incidence communities inside low-burden countries?", we collected data for the following variables:

1. Title
2. Number of people reached
3. Authors
4. Number of people with presumptive TB identified
5. Year
6. Number of people who required referral
7. Publication Year
8. Number of people evaluated for TB disease
9. Country of Screening
10. ATB cases detected N in Total
11. Year of screening
12. Percentage of microbiologically confirmed ATB Cases
13. Migrants
14. if paper concerns both ATB + LTBI
15. Refugees
16. specify how many ATB cases diagnosed/number of LTBI cases detected
17. Children
18. Percentage of MDR/total number of ATB cases
19. Elderly
20. HIV
21. Prisoners
22. HCV
23. ATB only
24. HBV
25. ATB + LTBI
26. Diabetes
27. NNS
28. Others
29. Questionnaire + CXR + Micro
30. Screened positive: Proportion of people presumed to have TB among those screened (C/B)
31. Questionnaire + TST/IGRA + CXR + Micro
32. Diagnostic delay
33. CXR for everyone eligible
34. Linkage to care: the proportion initiating TB treatment among those diagnosed (F/E)
35. TST/IGRA + CXR+ Micro
36. Treatment success: the proportion of people who successfully complete TB treatment among those who initiated treatment
37. Other
38. Total costs
39. Costs analysis perspective used
40. Cost PPS
41. Incremental C-E Ratio (ICER): Money per Case
42. Costs per group
43. ICER Ratio: Money per QALY (Quality Adjusted Life Years)
44. Cost per TB case detected/QALY/prevented
45. ICER: Money per TB case avoided"
46. Dominant Strategy(ies)
47. Real Costs
48. Dominated Strategy(ies).
49. Ignored Costs

Risk of Bias

We used Risk Of Bias In Non-randomised Studies - of Interventions (ROBINS-I) tool to evaluate the risk of bias in the studies included [10]

Table 1: ROBINS-I assessment

**Country**	**Risk of bias**	**Reference**
Belgium	Serious	[1]
Canada	Moderate	[2]

Germany	Moderate	[3]
Italy	Moderate	[4]
United Kingdom	Moderate	[5]
United Kingdom	Moderate	[6]
United Kingdom	Moderate	[7]
United Kingdom	Moderate	[8]
Canada	Low risk	[9]

Currency Conversion

Currencies converted using: https://eppi.ioe.ac.uk/costconversion/default.aspx
(1) All currencies were converted to the same currency in 2023
(2) Results were converted to Euro 2023, using Spain as a reference country
In both cases IMF for used as a source.

**Country**	**Year of the original Currency**	**Converted to Euro currency of Spain 2023**	**Reference**
Belgium	2014	Yes	[1]
Canada	2019	Yes	[2]
Germany	2019	Yes	[3]
Italy	2017	Yes	[4]
United Kingdom	2019	Yes	[5]
United Kingdom	2010	Yes	[6]
United Kingdom	2010	Yes	[7]
United Kingdom	2016	Yes	[8]
Canada	2010	Yes	[9]

References

1. Smit GSA, Apers L, De Onate WA, Beutels P, Dorny P, Forier AM, et al. Cost-effectiveness of screening for active cases of tuberculosis in Flanders, Belgium. Bull World Health Organ 2017;95:27–35. https://doi.org/10.2471/BLT.16.169383.
2. Uppal A, Nsengiyumva NP, Signor C, Jean-Louis F, Rochette M, Snowball H, Etok S, Annanack D, Ikey J, Khan FA, Schwartzman K. Active screening for tuberculosis in high-incidence Inuit communities in Canada: a cost-effectiveness analysis. Can. Med. Assoc. J. 2021 Nov 1;193(43):E1652-E1659. https://doi.org/10.1503/cmaj.210447
3. Wahedi K, Biddle L, Bozorgmehr K. Cost-effectiveness of targeted screening for active pulmonary tuberculosis among asylum-seekers: A modelling study with screening data from a German federal state (2002-2015). PLoS One. 2020;15(11):e0241852. https://doi.org/10.1371/journal.pone.0241852
4. Goscé L, Girardi E, Allel K, Cirillo DM, Barcellini L, Stancanelli G, et al. Tackling TB in migrants arriving at Europe's southern border. Int. J. Infect. Dis. 2021;113:S28–32. https://doi.org/10.1016/j.ijid.2021.02.103.
5. Capocci SJ, Sewell J, Smith C, Cropley I, Bhagani S, Solamalai A, et al. Cost effectiveness of testing HIV infected individuals for TB in a low TB/HIV setting. J. Infect. 2020;81:289–96. https://doi.org/10.1016/j.jinf.2020.05.055.
6. Pareek M, Bond M, Shorey J, Seneviratne S, Guy M, White P, et al. Community-based evaluation of immigrant tuberculosis screening using interferon γ release assays and tuberculin skin testing: observational study and economic analysis. Thorax. 2013;68(3):230-239. https://doi.org/10.1136/thoraxjnl-2011-201542
7. Jit M, Stagg HR, Aldridge RW, White PJ, Abubakar I. Dedicated outreach service for hard to reach patients with tuberculosis in London: Observational study and economic evaluation. *BMJ*. 2011;343:d5376. doi: https://doi.org/10.1136/bmj.d5376
8. Cavany SM, Vynnycky E, Anderson CS, Maguire H, Sandmann F, Thomas HL, et al. Should NICE reconsider the 2016 UK guidelines on TB contact tracing? A cost-effectiveness analysis of contact investigations in London. Thorax. 2019;74(2):185-193. https://doi.org/10.1136/thoraxjnl-2018-211662

9. Verma G, Chuck AW, Jacobs P. Tuberculosis screening for long-term care: A cost-effectiveness analysis. Int J Tuberc Lung Dis. 2013;17(9):1170-1177. https://doi.org/10.5588/ijtld.12.0934
10. Sterne J A, Hernán M A, Reeves B C, Savović J, Berkman N D, Viswanathan M et al. ROBINS-I: a tool for assessing risk of bias in non-randomised studies of interventions. BMJ 2016; 355:i4919 https://doi.org/10.1136/bmj.i4919
